# Editorial: Magnetic Resonance Imaging for Radiation Therapy

**DOI:** 10.3389/fonc.2020.00483

**Published:** 2020-04-15

**Authors:** Ning Wen, Yue Cao, Jing Cai

**Affiliations:** ^1^Department of Radiation Oncology, Henry Ford Health System, Detroit, MI, United States; ^2^Department of Radiation Oncology, University of Michigan, Ann Arbor, MI, United States; ^3^Department of Health Technology and Informatics, Hong Kong Polytechnic University, Kowloon, Hong Kong

**Keywords:** multiparametric MRI, synthetic CT (sCT), deep learning, MR Linac, radiomics analysis

Since the introduction of magnetic resonance imaging (MRI) to radiation therapy (RT), it has increasingly been adopted in RT treatment planning for target and organ-at-risk (OAR) definition due to superior soft tissue contrasts. Recently, the roles of MRI in RT have been advanced to tumor delineation in a multiparametric format, MRI-based treatment planning and dose calculation, MRI-guided treatment delivery, and outcome assessment using quantitative imaging metrics or radiomic features. These advancements are due to the development of dedicated MRI simulators, integration of MRI scanners with radiation treatment platforms, as well as technical developments and applications of 4D-MRI, tumor tracking, adaptive planning, and treatment response evaluation. The advancement of machine learning and artificial intelligence also brings about tremendous opportunities to transform the application of MRI in RT.

This collection includes 16 articles that cover the following themes:

- Target volume delineation and treatment planning workflow using MRI as the primary imaging modality

Multiparametric MRI (mp-MRI), a combination of morphologic and functional imaging modalities, has shown the potential to increase the accuracy of tumor detection, localization, and characterization of cancer aggression. Integration of mp-MRI techniques into RT offers enormous opportunities to individualize RT adaptation based upon the individual patient's response to treatment.

MR spectroscopy imaging (MRSI) can describe the metabolism of different tissues. However, the spatial resolution is limited due to the very low concentration of the metabolites in tissues. Iqbal et al. developed a densely connected U-Net to create super resolution spectroscopic images by training the T1 weighted images (T1WI) and the low-resolution ^1^H MR spectroscopic images together. They showed that the ^1^H spectra were maintained on retrospective *in vivo* data.

Lee et al. performed a volumetric and voxel-wise analysis of the dominant intraprostatic lesions (DIL) defined from T2-weighted imaging (T2WI), diffusion weighted imaging (DWI), and dynamic contrast enhanced (DCE) imaging respectively. The correlation was further classified according to tumor location and Gleason grade group. The data suggested that constructing a Boolean sum volume that incorporated T2WI and apparent diffusion coefficient (ADC) maps were reasonable for delineating the DIL on mp-MRI. The value of adding information provided by K^trans^ maps remains investigational due to the repeatability and consistency of DCE scans. The interobserver variability also indicated the need to develop a consensus guideline on DIL delineation using mp-MRI.

- Techniques to generate synthetic CT from MRI and the clinical implementation of MRI only workflow

Substantial interest has developed around generating synthetic CT (sCT) from MRI in order to use MRI as the only, or primary, imaging modality in the RT workflow. Different methods have been introduced to create sCT images using bulk density, atlas-based, or voxel-based approaches. Deep machine learning algorithms such as a U-Net or Generative Adversarial Network can learn image features among different imaging modalities and have great potentials to generate highly accurate synthetic images.

Choi et al. used a bulk anatomical density approach to develop a method for patient-specific quality assurance for MRI-only prostate RT. The three-class model (bone, muscle, and fat) provided accurate dose calculations for verifying sCT for clinical use in MRI-only workflows. The model has currently been implemented as a quality assurance method in a multi-center trial of prostate stereotactic RT that includes an MRI-only study.

Gupta et al. used a 3-channel U-Net trained on aligned MRI and CT pairs in sagittal planes to generate sCT images. The three channels represented Hounsfield Unit (HU) ranges of voxels containing air, soft tissue, and bone, respectively. The improved soft tissue contrast of sCT was proved with low mean absolute error difference between sCT and actual CT. The improved image quality was also beneficial for the online image registration with cone beam CT.

Wang et al. generated sCT from T2WI of nasopharyngeal carcinoma patients using a 2D U-net algorithm. The deep U-net with 23 convolutional layers was used to generate sCT. The soft tissue, nasal bone, bone marrow, and the interface between bones and soft tissues were carefully evaluated.

Greer et al. described a multi-center study for the implementation of an MRI-only prostate workflow. A sCT was created using an atlas-based method from whole pelvic T2WI scans with an isotropic 1.6 mm voxel. A CT scan was acquired subsequent to MRI only plan approval for patient specific quality assurance. The 3D gamma was calculated to evaluate the dose difference between sCT and actual CT, and gold fiducial marker positions were used to evaluate the image registration accuracy between sCT and actual CT. All 25 patients recruited were treated with MRI only workflow.

Mittauer et al. developed an MRI-guided online adaptive radiotherapy (MRgoART) procedure for palliative care in RT. The electron density information was incorporated with either a bulk density override or deformable image registration of diagnostic CT to the MRI. The plan quality and treatment delivery efficiency were superior than the conventional method. Excellent clinical outcomes were observed and were in line with historical and sampled controls.

- Quantitative analysis of morphological and functional MRI and their applications in treatment response assessment

Quantitative MRI can reflect tissue characteristics. Imaging biomarkers from functional MRI can have prognostic and predictive values for progression free survival, overall survival, and distant metastases etc. Radiomic features, which are defined as the post-processing for extraction of textural information from medical images, can provide tremendous information to analyze and characterize the properties of tumor tissues and their physiological and pathological stages.

In this collection, Cao et al. analyzed MRI-derived gross tumor volume, blood volume, and ADC from pre-treatment and mid-treatment, as well as pre-treatment FDG PET metrics for locally advanced head and neck cancer (HNC) treated with chemoradiation. The mean ADC values from pre-RT and its change rate mid-treatment were significant higher and lower in p16– than p16+ locally advanced HNC tumors, respectively. These biomarkers had predictive values and compared favorably with FDG-PET imaging markers.

van Schie et al. analyzed T2 and ADC changes during treatment and compared patients with and without hormonal therapy, as the hypoFLAME trial patients received ultra-hypofractionated prostate radiotherapy with an integrated boost to the tumor in 5 weekly fractions. Significant ADC changes were observed in the tumor in patients without hormonal therapy. Such early response measured with quantitative MRI holds the potential to predict clinical outcome and guide treatment adaptation.

Bagher-Ebadian et al. extracted discriminant radiomic features in the real radiomics-feature space and the latent-variable space from mp-MRI for prostate cancer. These features were used to construct an artificial neural network to classify the DIL from normal prostatic tissues.

Li Z. et al. analyzed pre-treatment T1WI, T2WI, and DWI for esophageal squamous cell carcinoma patients undergoing concurrent chemoradiotherapy and identified the ADC texture features that can be used to predict the overall survival.

Yu et al. also analyzed pre-treatment T1WI, T2WI to identify tumoral radiomic features that were used to predict patient eligibility for adaptive radiotherapy in advanced nasopharyngeal carcinoma (NPC) patients.

Considering post-treatment changes are often highly heterogeneous, including cellular tumor, fat, necrosis, and cystic tissue compartments, evaluation of the tumors defined using pre-treatment images could be limited to predict treatment response. Blackledge et al. studied 8 commonly used supervised machine-learning algorithms for tissue classification of mp-MRI of soft tissue sarcoma to quantify post-RT changes. Five out of eight algorithms achieved similar performance. Of the five methods, the Naïve-Bayes classifier was chosen for further investigation due to its relatively short training and prediction times.

- Development of MR-Linac

Recent commercial developments of MRI-guided RT platforms provide great opportunities for direct imaging guidance, tumor/OAR tracking during RT, and treatment adaptation. Two systems have received CE and/or FDA 510 k clearance so far: Unity (Elekta, Sweden) and MRIdian system (Viewray, USA). The Australian MRI-linac system is at the research prototype stage and has an inline orientation, with radiation beam parallel to the main magnetic field. Such inline design can help minimize magnetic field influence on dose deposition. Jelen et al. developed methods to quantify dosimetric characteristics of the Australian MRI-linac system.

- Review papers

There are two review papers in this collection. As MRI-guided RT, including adaptive RT, have advanced in the field, the community needs to develop protocols on how to make clinical decisions with funneling MRIgRT data. Kiser et al. discussed the challenges of interpretability and reproducibility of MRI data, the complexity of a variety of MR sequences, and the corresponding impacts on RT workflow, such as synthetic CT generation, image fusion, dose calculation, and prognostic values using radiomic features.

Li G. et al. reviewed two 4D-MRI techniques—respiratory-correlated (RC) and time-resolved (TR) 4D-MRI. The RC-4DMRI was reconstructed to provide one-breathing-cycle motion, while the TR-4DMRI provided an adequate spatiotemporal resolution to assess tumor motion and motion variation. Both techniques were also discussed in the context of their clinical applications in radiotherapy.

Benefitting from advanced technologies of synthetic CT techniques and MR Linacs, the MR-solely RT workflow has been rapidly evolving and has been clinically implemented widely. It has potential to improve the therapeutic gains for certain disease sites through dose escalation with better tumor delineation and motion management. Randomized clinical trials have been promoted to investigate the effects of dose escalation on normal tissue toxicity, quality of life, as well as overall survival and local control for prostate cancer, locally advanced pancreatic cancer, etc. As MRI is playing an increasingly essential role in RT, opportunities arise to incorporate functional imaging into RT workflow. Considering the response of the ADC maps to radiation dose and relatively robust protocol for DWI acquisition, DWI-derived biomarkers have strong potentials for tumor delineation and response assessment, as evidenced in a series of articles published in this collection.

## Author Contributions

NW, YC, and JC are the coeditors for this Research Topic.

### Conflict of Interest

The authors declare that the research was conducted in the absence of any commercial or financial relationships that could be construed as a potential conflict of interest.

